# Premarital Screening of Beta Thalassemia Minor in north-east of Iran

**Published:** 2013-01-22

**Authors:** H Hashemizadeh, R Noori

**Affiliations:** 1 Department of Nursing, Quchan Branch, Islamic Azad University, Quchan, Iran.; 2 Department of Midwifery, Quchan Branch, Islamic Azad University, Quchan, Iran.

**Keywords:** Prevalence, beta-Thalassemia, Premarital Examinations

## Abstract

**Background:**

Beta thalassemia is a preventable disease. Iran has about 20,000Patients who are homozygote for β-thalassaemia and 3,750,000 carriers. The aim of this study was to determine the prevalence of beta thalassemia minor among men who underwent premarital screening in Quchana city in Khorasan Razavi region of Iran

**Materials and Methods:**

This research is a descriptive cross-sectional study. From 2010 to 2011, all participants (1000) under marriage coming to health center of Quchan underwent routine mandatory tests. Participants were considered to have beta-thalassemia minor on the condition that hey had a mean corpuscular volume (MCV) <80fl and a mean corpuscular hemoglobin (MCH) <27 pg and a hemoglobin A2 level >3.5%. Venous blood was taken into an EDTA tube and the complete blood count and red blood cell indices were measured with a Coulter automated cell counter. Electrophoresis was performed on cellulose acetate.

**Results:**

Mean and SD of hemoglobin, MCV and MCH were 16±2.9, 91±4 and 28.4±2, respectively. Hemoglobin A2 Higher than 3.5 percent was reported as 3.5%.The prevalence of beta-thassemia minor with high hemoglobin A2 and microcytic hypochromic anemia was 3.5% (P-value).

**Conclusion:**

In countries with high prevalence of hemoglobinopathies, a premarital screening program is helpful for identification and prevention of high-risk marriages. Detecting carrier couples with premarital screening program is an effective way of controlling thalassemia major.

## Introduction

Beta-thalassemia is one of the most common genetic diseases in Iran. Iran has about 20,000 Patients who are homozygote for β-thalassaemia and 3,750,000 carriers (1). Thalassemia is caused by impaired production of either the alpha or beta hemoglobin chain, and is accordingly classified as alpha or beta thalassemia. Alpha thalassemia is relatively rare, whereas beta thalassemia is quite common in certain parts of the world. Beta thalassemia clinically presents as thalassemia trait (thalassemia minor) or thalassemia major. Compared with patients with thalassemia major (HbA2 2%, HbF 98%, HbA Nil) who present with severe illness and require periodic blood transfusions, patients with thalassemia trait (HbA2 >3.5%, HbF Nil) are clinically well, and are usually one detected through routine blood testing. However, the children of such patients could inherit the disease if the patient's partner also has the beta thalassemia trait (2). Information about the prevalence of thalassemia in Iran is not clear, but studies have reported that thalassemia is relatively common genetic disorders in this part of the world. A number of studies was conducted in Iran, demonstrated that the prevalence of these diseases varied significantly in different parts of Iran, with the highest prevalence in the Caspian Sea, and Persian Gulf. Thalassemia is common, incurable, autosomal recessive inheritable haemoglobinopathy that cause significant morbidity and mortality and impose a heavy financial burden on society. A simple blood test before marriage can easily detect carriers of these diseases, to inform couples about their chances of producing affected children and ensure they receive appropriate advice. In the early 1970s, some premarital screening programs have become widely accepted; so many countries have made them mandatory (3). Thalassemia premarital screening program was started from 1997 in Iran .The thalassemia prevention program in Iran is based upon premarital screening of *β*-thalassaemia couples (carriers) in order to encourage them to participate in counseling and prenatal diagnosis (PND) (4).

In this program, red cell indices are checked. If mean corpuscular hemoglobin (MCH) <27 pg or mean corpuscular volume (MCV) <80fl (cut off values) were found in both couples, hemoglobin A2 concentrations will be measured. If it is confirmed as characteristic for minor *β*-thalassaemia (Hb A2 > 3.5), the couples were referred for counseling. But when both partners have red cell indices lower than the cut off values with an Hb A2 concentration in the normal range, they are considered as suspect. Then, they must undergo some additional stages including a course of iron therapy and subsequent recheck of the indices with or without molecular studies (5).

In reference hematology books and also in literature, different cut off values have been mentioned for MCH and MCV which might be due to the difference in the characteristics of the studied populations, such as age and race (5, 6). In this study, MCV cut-off values suggested 80 fl and MCH cut –off values suggested 27pg.

Premarital testing is not acceptable in some communities for various legal and religious reasons, and other educational and cultural factors may prevent some married couples following the advice given by Counselors. The success of these programs depends on adequate religious support, governmental policy, education and Counseling (7). The prevalence of inherited blood disorders in certain parts of the world is high, including autosomally inherited haemoglobinopathies, thalassaemia and sickle cell disease. Premarital screening aims to identify carriers of the haemoglobin disorders, in order to assess the risk of having children with a severe form of disease. 

The couple can then choose whether or not to have an affected child (8). Premarital thalassaemia screening was first carried out in 1975 by Silvestroni and in Latium, Italy, as part of a school prevention program (9).

This research was conducted to determine the prevalence of minor β-thalassaemia based on RBC indices in 1000 men under marriage referred to Quchan (north east of Iran) city health center.

## Materials and Methods

This research is a descriptive cross-sectional study. From 2010 to 2011, all men (1000) who intended to get married coming to health center of Quchan underwent routine mandatory tests. Subjects were considered to have beta-thalassemia trait if they had MCV <80 fl and MCH <27 pg and a hemoglobin A2 level >3.5%. Venous blood was taken into an EDTA tube and the complete blood count and red blood cell indices were measured with a Coulter automated cell counter on the same day of hemoglobin collection. Electrophoresis was performed on cellulose acetate. For all men, red cell indices were checked. If MCH <27 pg or MCV <80fl were found, hemoglobin A2 concentrations were measured. Three ml of blood for laboratory studies was collected. Sysmex KX-21N was used for cell counting. For determination of Hb A2, manual kit bio system by column Chromatography was used.

**Statistical analysis**

Patients’ data was recorded in a questionnaire and Statistic analysis was performed with SPSS v.16. Finally, the frequency of Hb, MCV, MCH and Hb A2 was calculated.

## Results

The mean age of study group was 23.5±2.5 years. The level of Hemoglobin in Most of them was 14 gr / dl(62.6%),the level of MCV in most of them was 90 Fl(73.61%).39.17% had MCH at level of 28 pg and 45% had MCHC at level of 30 g / dl. Mean and SD of hemoglobin, MCV, MCH were 16 ± 2.9, 91 ± 4 and 28.4 ± 2, respectively. Hemoglobin A2 more than 3.5 percent was reported as 3.5%.

MCH ≥28 pg, MCV≥81fl and hemoglobin A2≤ 3.4 % were found in 90.5%, 89 % and 96.5 % of patients.

**Table I:** The result of premarital screening program.MCV: mean corpuscular volume, MCH: mean corpuscular hemoglobin.

**Variable**	**Mean±SD**	**Min**		**Max**
**Hemoglobin**	16 ± 2.9	8		18
**MCV**	91 ± 4	65		110
**MCH**	28.4 ± 2	22		30
**HbA2**	2.5±0.2	2		7

**Table II:** The frequency of Hemoglobin. Most of the sample had hemoglobin between 14-17 g/dl.

**Hemoglobin**	**Patient number**	**Patient percent**
**≤13**	100	10
**14-17**	860	86
**18≥**	40	4
**Total**	1000	100

**Table III:** - Frequency of beta thalassemia minor in other country. Sardinia has the most population for beta thalassemia minor.

**Location**	**Frequency of β-thalassemia minor**
**Sardinia(17)**	11-34%
**Sicily(18)**	10%
**Greece(18)**	5-15%
**Iran(16)**	4-10%
**Turkey %(**19**)**	2.1%
**Saudi Arabia** **(**21**)**	3.22%

**Table IV:** Frequency of beta thalassemia minor in Iran. The highest prevalence of beta thalassemia minor was reported in Khoozestan  and Mazandaran province.

**Location**	**Frequency of β-thalassemia minor**
**Isfahan(16)**	8%
**Fars (16)**	8-10%
**Kashan(25)**	3.22 %
**Kerman(26)**	2.6%
**sanandaj(27)**	3.6 %
**SistanBaluchestan(27)**	7.5%
**Birjand(28)**	1%
**Khoozestan(29)**	10%
**Mazandaran(30)**	10%

## Discussion

Yet, not all screening programs have shared this degree of success. Some very strong social factors influence the acceptability of preventive programs, not least among them religious beliefs, cultural norms, traditions, literacy and education level, govern mental policies and the attitudes of individual couples. Experiences from some Islamic countries indicate that the way in which some individuals misinterpret their religion, creates a significant obstacle to the success of screening programs in Muslim communities (10). MCV and MCH are suitable for epidemic screening in a large population, physical examination and premarital check-up. Hb electrophoresis and thalassemia gene diagnosis are recommended for subjects with positive MCV and MCH indexes (11). Detecting carrier couples with premarital screening program is an effective way of controlling thalassemia major (12). Premarital screening markedly reduced the number of at-risk marriages (13).

In countries with a high prevalence of hemoglobinopathies, a premarital screening program is helpful for identification and prevention of high-risk marriages. 

The current article reports the impact of one year of premarital screening on the prevalence of β-thalassemia in Quchan. With a 3.5% prevalence of beta-thalassemia trait in premarital status; future comprehensive programs are needed to know the actual prevalence of beta-thalassemia in Quchan. Also determine the prevalence of beta-thalassemia minor in the other areas of the Iran to perform genetic counseling and perinatal diagnosis seems essential.

Thalassemia is found in some 60 countries with the highest prevalence in the Mediterranean region, parts of North and West Africa, the Middle East, the Indian subcontinent, southern Far East and southeastern Asia, together composing the so-called thalassemia belt. In western countries, thalassemia affects mostly individuals whose ancestry are traceable to a high prevalence areas (14, 15) As an example, there are around 1,000 cases of beta thalassemia major in the United States, most of whom are descendants of Mediterranean, Asian Indian, South Asian, or Chinese ancestors (14). This figure is even less than half of the number of beta thalassemic patients in Fars Province, a region with only 120,000 km2 large in southern Iran (16).

About 150 million people worldwide carry beta thalassemia genes. The genes are particularly prevalent in Italy and Greece. Other regions with the high gene frequency are Sardinia (11 to 34%) (17), Sicily (10%) (18), Greece (5 to 15%) (18) and Iran (4 to 10%) (16). 

A research on 48,126 persons in Turkey showed that the prevalence of β-thalassemia trait and sickle cell anemia trait 2.1% and 0.5% (19).

Another research in turkey on 19,804 subjects showed the prevalence of β-thalassemia trait 2.6% (20). The prevalence of beta-thalassemia trait in premarital screening program in Al-Hassa, Saudi Arabia was 3.4% (21).

Another research on Saudi Arabia was showed the prevalence of β-thalassemia trait and sickle cell anemia trait 1.8% and 4.5% (22).

A research on 488,315 individuals screened in Saudi Arabia, Showed that 4.20% had sickle cell trait, 0.26% had sickle cell disease, 3.22% had thalassemia trait, and 0.07% had thalassemia disease (23).

A total of 88,888 people were screened in Kocaeli, Turkey. The frequencies of beta thalassemia trait and sickle cell anemia trait were 0.89% and 0.05%, respectively. The frequency of couples with high-risk of having a sibling with homozygous hemoglobinopathy was 0.01%.The prevalence of beta-thalassemia trait and sickle cell anemia trait was quite low and reflects the frequency in eastern and northern Anatolia and migration to Kocaeli from these geographic regions ( 24).

Iran, a country with an area of 1,648,000 km^2^, like many other countries in the region has a large number of major thalassemia patients. Alfa thalassemia is very rare in Iran. The gene frequency of beta thalassemia, however, is high and varies considerably from area to area, having its highest rate of more than 10% around the Caspian Sea, and Persian Gulf. The prevalence of the disease in other areas is between 4% and 8%. In Isfahan, a city built around the river Zayandeh-Rood in the central part of Iran, the frequency rises again to about 8%. In the Fars Province, in southern Iran, the gene frequency is also high and reaches 8 to 10% (16).In Kerman ,Kashan, sanandaj, SistanBaluchestan, Birjand , khoozestan and Mazandaran were 2.6 %, 3.22 %3.6 % ,7.5%,1% ,10% and 10% respectively ( 25,26, 27 , 28, 29, 30).

Screening programs and prenatal diagnosis in cyprus, Greece and Italy has accomplished a 100% achievement and the birth prevalence of homozygote beta thalassemia has reached zero. In Iran, before the start of this screening program, we had a new case of thalassemia every 6 hours. Nearly 20,000 homozygote beta thalassemias are living in Iran. By applying the screening program, the birth prevalence of beta thalassemia major has declined from 0.253 for every 100 births in 1995 to 0.082 for every 100 births in 2004.It needs to be evaluated in order to reach the zero in beta thalassemia birth like other countries such as Greece and Cyprus (1).

## Conclusion

In countries with a high prevalence of hemoglobinopathies, a premarital screening program is helpful for identification and prevention of high-risk marriages. Detecting carrier couples with premarital screening program is an effective way of controlling thalassemia major.
